# Population-Specific Genetic and Expression Differentiation in Europeans

**DOI:** 10.1093/gbe/evaa021

**Published:** 2020-02-06

**Authors:** Xueyuan Jiang, Raquel Assis

**Affiliations:** e1 Huck Institutes of the Life Sciences, Pennsylvania State University, University Park, PA 16802; e2 Department of Biology, Pennsylvania State University, University Park, PA 16802; e3 Department of Computer and Electrical Engineering and Computer Science, Florida Atlantic University, Boca Raton, FL 33431; e4 Institute for Human Health and Disease Intervention, Florida Atlantic University, Boca Raton, FL 33431

**Keywords:** human evolution, population genetics, expression divergence, genetic divergence

## Abstract

Much of the enormous phenotypic variation observed across human populations is thought to have arisen from events experienced as our ancestors peopled different regions of the world. However, little is known about the genes involved in these population-specific adaptations. Here, we explore this problem by simultaneously examining population-specific genetic and expression differentiation in four human populations. In particular, we derive a branch-based estimator of population-specific differentiation in four populations, and apply this statistic to single-nucleotide polymorphism and RNA-seq data from Italian, British, Finish, and Yoruban populations. As expected, genome-wide estimates of genetic and expression differentiation each independently recapitulate the known relationships among these four human populations, highlighting the utility of our statistic for identifying putative targets of population-specific adaptations. Moreover, genes with large copy number variations display elevated levels of population-specific genetic and expression differentiation, consistent with the hypothesis that gene duplication and deletion events are key reservoirs of adaptive variation. Further, many top-scoring genes are well-known targets of adaptation in Europeans, including those involved in lactase persistence and vitamin D absorption, and a handful of novel candidates represent promising avenues for future research. Together, these analyses reveal that our statistic can aid in uncovering genes involved in population-specific genetic and expression differentiation, and that such genes often play important roles in a diversity of adaptive and disease-related phenotypes in humans.

## Introduction

Human phenotypes vary widely across the globe. In particular, geographically separated populations often differ in skin pigmentation (Loomis 1967), hair color (Rees 2003), tooth morphology (Scott and Turner 1997; Hanihara and Ishida 2005), surface area to body mass ratio (Katzmarzyk and Leonard 1998), and predisposition to diseases (Frank 2004). Much of this phenotypic variation is thought to have arisen due to a diversity of selective pressures experienced as early humans peopled the world and encountered novel environments (Sabeti et al. 2002; Voight et al. 2006), food sources (Sabeti et al. 2002), and pathogens (Diamond 2002; Jobling et al. 2013). As a result, uncovering the genetic targets of phenotypic differentiation among human populations is critical both for understanding past human adaptations (Sabeti et al. 2002) and for advancing future biomedical research (Jorde et al. 2001; Akey et al. 2004).

Due to the abundance of whole-genome sequence and polymorphism data for many human populations (Cann et al. 2002; International HapMap 3 Consortium 2010; 1000 Genomes Project Consortium 2015), much work in the past several years has focused on elucidating and understanding genetic differentiation that occurred during human evolution (Li et al. 2008; Pickrell et al. 2009; Field et al. 2016). A common summary statistic for estimating genetic distances between two populations is the fixation index, $F_{\mathrm{ST}}$ (Wright 1951), which has been used to infer human demographic history (Hinds et al. 2005; Holsinger and Weir 2009; Keinan et al. 2009; Patterson et al. 2012; 1000 Genomes Project Consortium 2015) and to identify loci that may be targets of natural selection (Bowcock et al. 1991; Akey et al. 2002; Bersaglieri et al. 2004). However, because $F_{\mathrm{ST}}$ is a pairwise metric, it cannot identify the directionality of genetic differentiation nor be used as sole evidence for natural selection (Yi et al. 2010). To address this issue, Yi et al. (2010) developed the Population Branch Statistic (PBS), a summary statistic that utilizes pairwise $F_{\mathrm{ST}}$ values among three populations to quantify genetic differentiation along each branch of their corresponding three-population tree. Genes with large PBS values on one branch represent loci that underwent population-specific genetic differentiation consistent with relaxed selective constraint or positive selection (Yi et al. 2010). PBS has been applied to corroborate previously established targets of selection, including genes associated with skin pigmentation (Lamason et al. 2005) and dietary fat sources (Mathias et al. 2012), as well as to identify novel candidates for high-altitude adaptation in Tibetans (Yi et al. 2010).

However, because natural selection acts on phenotypes, analysis of genetic data only enables assessment of its indirect effects. For this reason, it may be advantageous to study selection more directly by exploiting the recent availability of RNA-seq data for several human populations (Lappalainen et al. 2013). Specifically, phenotypic evolution is thought to often occur through modifications in gene expression (King and Wilson 1975; Wang et al. 1996; Wray et al. 2003; Carroll 2005, 2008; Raj et al. 2010). Thus, studying gene expression differentiation among human populations may increase power for identifying loci underlying population-specific phenotypes. Indeed, like genetic differentiation, gene expression levels vary considerably across human populations (Cheung et al. 2005; Stranger et al. 2007) and often reflect population structure (Brown et al. 2018). Moreover, human genes with large PBS values are enriched for expression quantitative trait loci (Quiver and Lachance 2018).

In the present study, we simultaneously explore population-specific genetic and expression differentiation in four human populations: the Toscani in Italia (TSI), British in England and Scotland (GBR), Finnish in Finland (FIN), and Yoruba in Nigeria (YRI). For these analyses, we employ single-nucleotide polymorphism (SNP; 1000 Genomes Project Consortium 2015) and RNA-seq (Lappalainen et al. 2013) data from each population. First, we use $F_{\mathrm{ST}}$ (Wright 1951) and its analog for estimating quantitative trait differentiation, $P_{\mathrm{ST}}$ (Leinonen et al. 2006), to quantify and examine genome-wide patterns of genetic and expression differentiation in the four human populations. Next, we adapt the approach of PBS (Yi et al. 2010) to $P_{\mathrm{ST}}$, and extend its computation to a four-population tree, enabling us to estimate both genetic and expression differentiation in each of the four human populations. Last, we apply this branch-based statistic to study population-specific genetic and expression differentiation, and uncover candidate genes and functional modules underlying adaptation in TSI, GBR, and FIN populations.

## Results

### Genome-Wide Patterns of Genetic and Expression Differentiation in Four Human Populations

A first goal of our study was to estimate genetic and expression differentiation among TSI, GBR, FIN, and YRI populations. To address this problem, we used SNP data (1000 Genomes Project Consortium 2015) to calculate the $F_{\mathrm{ST}}$ (Wright 1951), and RNA-seq data (Lappalainen et al. 2013) to calculate the $P_{\mathrm{ST}}$ (Leinonen et al. 2006), of every gene between each pair of the four human populations. We calculated $F_{\mathrm{ST}}$ using Hudson’s formula (Hudson et al. 1992) and computed the ratio of averages to minimize bias (Reynolds et al. 1983; Weir and Cockerham 1984; International HapMap 3 Consortium 2010; Bhatia et al. 2013; see Materials and Methods for details). Due to environmental effects on $P_{\mathrm{ST}}$, we followed the approach of Leinonen et al. (2006) in calculating $P_{\mathrm{ST}}$ under two contrasting scenarios: one in which environmental and nonadditive genetic effects account for half of the observed expression variation ($h^{2}=0.5$), and a second in which only additive genetic effects contribute to the observed expression variation ($h^{2}=1$; see Materials and Methods for details). Examinations of Pearson’s linear (r) and Spearman’s nonlinear (ρ) correlations revealed small ($∼10^{-2}$) but significantly positive relationships between $F_{\mathrm{ST}}$ and $P_{\mathrm{ST}}$ in TSI–FIN, TSI–YRI, GBR–YRI, and FIN–YRI population pairs (supplementary tables 1 and 2, Supplementary Material online), consistent with previous observations that genetic and expression differentiation are weakly or moderately associated (Makova and Li 2003; Nuzhdin et al. 2004; Sartor et al. 2006; Assis and Bachtrog 2013, 2015; Hunt et al. 2013).

**Fig. 1.**
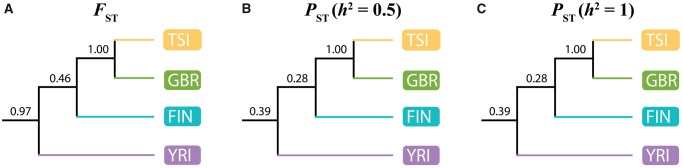
—Relationships among TSI, GBR, FIN, and YRI populations inferred from genome-wide patterns of genetic and expression differentiation. Population trees supported by the majority of gene trees constructed using (*A*) $F_{\mathrm{ST}}$, (*B*) $P_{\mathrm{ST}}$ with $h^{2}=0.5$, and (*C*) $P_{\mathrm{ST}}$ with $h^{2}=1$. Numbers indicate proportions of corresponding nodes in all gene trees (see Materials and Methods for details).

To explore genome-wide patterns of genetic and expression differentiation among the four human populations, we independently used $F_{\mathrm{ST}}\;$and $P_{\mathrm{ST}}$ to construct gene trees and then infer population trees supported by majorities of these gene trees (see Materials and Methods for details). Population trees inferred from $F_{\mathrm{ST}}$ and $P_{\mathrm{ST}}$ (with $h^{2}=0.5$ and $h^{2}=1$) have the same topology (fig. 1), indicating that there is consistency between relationships estimated from genome-wide patterns of genetic and expression differentiation despite their weak correlations with one another. Further, the topology of the inferred population trees recapitulates known relationships among these four populations, in that TSI and GBR are most closely related to one another, FIN is an outgroup to TSI and GBR, and YRI is an outgroup to all three European populations. These results mirror those from similar studies of $F_{\mathrm{ST}}$ (Hinds et al. 2005; Jakobsson et al. 2008; Li et al. 2008; Auton et al. 2009; Holsinger and Weir 2009; Keinan et al. 2009; Patterson et al. 2012; 1000 Genomes Project Consortium 2015), as well as findings that gene expression data often display population structure comparable to that of genetic data (Cheung et al. 2005; Stranger et al. 2007; Brown et al. 2018).

Yet, there is greater support for the inferred population tree when using $F_{\mathrm{ST}}$ (fig. 1*A*) than when using $P_{\mathrm{ST}}$ (fig. 1*B* and *C*) as input. This effect is not surprising, given the complex and dynamic nature of gene expression data. Specifically, gene expression levels can vary across space (e.g., cell type), time (e.g., age), and condition (e.g., disease). Additionally, the experimental methodology used to collect and quantify these data may influence expression levels as well. This contrasts with the relatively static nature of genetic data. Further, whereas our calculation of $F_{\mathrm{ST}}$ for a gene was often based on allele frequencies at multiple SNPs across the gene, our calculation of $P_{\mathrm{ST}}\;$for a gene was based on a single measurement. Therefore, differing levels of support observed for the inferred population trees may reflect higher accuracy and lower variance in estimating $F_{\mathrm{ST}}$ given the more representative and larger samples available for genetic data.

To investigate this effect, we examined the association between the number of SNPs in a gene and the difference between topologies of the gene tree constructed with $F_{\mathrm{ST}}$ and the population tree. In particular, if mismatches between gene trees constructed with $P_{\mathrm{ST}}\;$and the population tree are often due to the small sample size of expression data, then we also expect gene trees constructed with $F_{\mathrm{ST}}$ to be different from the population tree when the number of SNPs is small. To quantify the difference between each gene tree constructed with $F_{\mathrm{ST}}$ and the population tree, we used the Robinson–Foulds (RF) distance, which is the sum of the number of unique clades in the two trees being compared (Robinson and Foulds 1981). Here, RF = 0 when the tree topologies are identical, RF = 2 when there is one unique clade in each tree, and RF = 4 when the tree topologies are distinct. As hypothesized, there is an inverse relationship between RF and the number of SNPs, in that we tend to get RF = 0 when the number of SNPs is largest, RF = 2 when the number of SNPs is intermediate, and RF = 4 when the number of SNPs is smallest (supplementary fig. 1, Supplementary Material online; P<0.01 for all pairwise comparisons, two-sample permutation tests; see Materials and Methods for details). Hence, whereas genome-wide patterns of genetic and expression differentiation likely reflect true population relationships (fig. 1), gene-level values of $F_{\mathrm{ST}}$, and particularly of $P_{\mathrm{ST}}$, should be interpreted with caution.

### Estimation of Population-Specific Genetic and Expression Differentiation on a Four-Population Tree

**Fig. 2.**
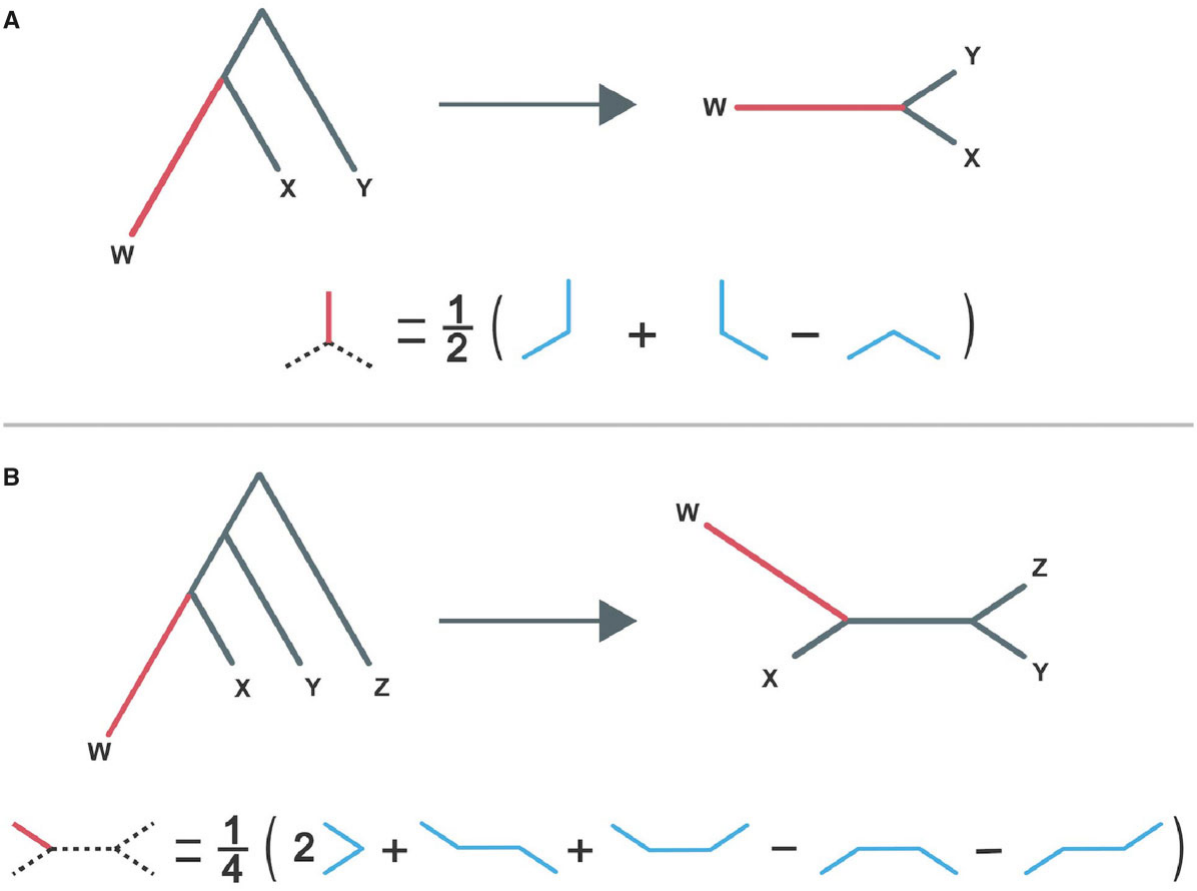
—Schematic for calculating the PBS value of a gene in population *W*. Depicted are scenarios in which population-specific differentiation of a gene has occurred in population *W* of a set of (*A*) three populations *W*, *X*, and *Y* and (*B*) four populations *W*, *X*, *Y*, and *Z*. In each case, population-specific differentiation results in elongation of external branch *W* (red). To estimate the length of external branch *W*, we unroot the tree (top of each panel) and apply the formula shown (bottom of each panel) to pairwise genetic ($F_{\mathrm{ST}}$) or expression ($P_{\mathrm{ST}}$) distances between populations. We can use an analogous approach to estimate lengths of other external branches.

Next, we sought to quantify population-specific genetic and expression differentiation of genes in each of the four human populations. For a three-population tree, population-specific genetic differentiation of a gene along each branch can be estimated with PBS (Yi et al. 2010; fig. 2*A*), which applies equation (11.20) in Felsenstein (2004) to $F_{\mathrm{ST}}$. In particular, considering the unrooted three-population tree shown in figure 2*A*, the PBS value of a particular gene in population *W* is estimated as PBS_*W*_$=\;\frac{1}{2}($*E_W_*_,__*X*_+*E_W_*_,__*Y*_–*E_X_*_,__*Y*_), where *E_W_*_,__*X*_, *E_W_*_,__*Y*_, and *E_X_*_,__*Y*_ denote the log-transformed $F_{\mathrm{ST}}$ between populations *W* and *X*, *W* and *Y*, and *X* and *Y*, respectively (Yi et al. 2010; see Materials and Methods for details). In a recent study, equation (11.20) in Felsenstein (2004) was also applied to expression distances between orthologous genes to estimate branch lengths corresponding to lineage-specific expression divergence on a three-species tree (Assis 2019). Analogously, by substituting $P_{\mathrm{ST}}$ for $F_{\mathrm{ST}}$ in the formula for PBS (Yi et al. 2010), we can obtain the PBS corresponding to gene expression differentiation in population *W* on the three-population tree. To distinguish between these two PBS in our study, we will refer to the calculation with $F_{\mathrm{ST}}$ as “genetic PBS,” and the calculation with $P_{\mathrm{ST}}$ as “expression PBS.”

To enable quantification of population-specific genetic and expression differentiation in four human populations, we extended the derivation of PBS to a four-population tree (fig. 2*B*). Henceforth, we will denote PBS as PBS_3_ when applied to a three-population tree (fig. 2*A*) and as PBS_4_ when applied to a four-population tree (fig. 2*B*). To derive PBS_4_, suppose that we have four populations *W*, *X*, *Y*, and *Z* that are related by the unrooted tree depicted in figure 2*B*. Then, we can compute four PBS_4_ values for a particular gene, one corresponding to its population-specific differentiation in each population. Because the PBS_4_ value for a gene in a population represents its differentiation that occurred in the lineage of that population, it can be estimated by the length of the external branch corresponding to the population. We can obtain the length of each external branch by first computing four distances: those between populations *W* and *X* (*E_W_*_,__*X*_), *W* and *Y* (*E_W_*_,__*Y*_), *X* and *Y* (*E_X_*_,__*Y*_), and *X* and *Z* (*E_X_*_,__*Z*_). Then, we can use these distances to compute the length of each external branch by following the schematic pictured in figure 2*B*. For example, the PBS_4_ value of the gene in population *W* is calculated as PBS_4,__*W*_$=\;\frac{1}{4}(2$*E_W_*_,__*X*_+*E_W_*_,__*Y*_+*E_W_*_,__*Z*_– *E_X_*_,__*Y*_–*E_X_*_,__*Z*_). Using this formula, we computed the genetic PBS_4_ and expression PBS_4_ of each gene in TSI, GBR, FIN, and YRI populations (supplementary tables 3–5, Supplementary Material online; see Materials and Methods for details).

### Population-Specific Genetic and Expression Differentiation of Genes with Copy Number Variations

**Fig. 3.**
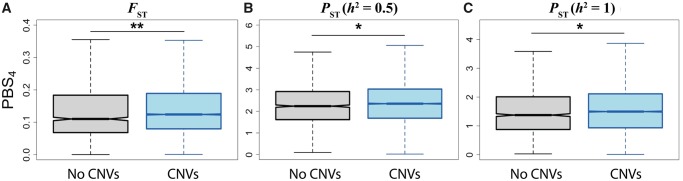
—PBS_4_ values of genes with CNVs. Distributions of (*A*) genetic PBS_4_ values calculated from $F_{\mathrm{ST}}$, (*B*) expression PBS_4_ values calculated from $P_{\mathrm{ST}}\;$with $h^{2}=0.5$, and (*C*) expression PBS_4_ values calculated from $P_{\mathrm{ST}}\;$with $h^{2}=1\;$of genes without (gray) and with (blue) CNVs. *P<0.05 and **P<0.001 (see Materials and Methods for details).

Gene duplications and deletions are key contributors to human genetic diversity (Sudmant et al. 2015). Moreover, because they are large-scale mutation events that may impact gene dosage, duplications and deletions have been implicated in numerous human diseases (Sebat et al. 2004; Kumar et al. 2008; Sharp et al. 2008; Weiss et al. 2008), as well as in adaptive events in many diverse species (Kaessmann 2010; Chen et al. 2013). For these reasons, genes harboring copy number variations (CNVs) are thought to be more frequently targeted by natural selection than those without CNVs (Freeman et al. 2006; Nguyen et al. 2006). Indeed, genes with CNVs often display signatures of adaptation (Sudmant et al. 2015), and fixation of duplications and deletions has been associated with natural selection in many species (Freeman et al. 2006; Nguyen et al. 2006; Han, Demuth, et al. 2009; Jiang and Assis 2017). Therefore, we hypothesized that genes with CNVs would have larger genetic and expression PBS_4_ values than genes without CNVs. To test this hypothesis, we compared the distributions of maximum PBS_4_ values of genes with and without known human CNVs larger than 50 bp (fig. 3; MacDonald et al. 2014; see Materials and Methods for details). As expected, both genetic and expression PBS_4_ values are significantly elevated in genes with CNVs (fig. 3; P<0.05 for all pairwise comparisons, two-sample permutation tests; see Materials and Methods for details). Though the magnitudes of the effects are modest, genes with CNVs also contain more SNPs than those without CNVs (P<0.001, two-sample permutation test; see Materials and Methods for details), which is expected to decrease their genetic PBS_4_ values (Yi et al. 2010). Taken together, these findings suggest that genes with CNVs tend to undergo increased population-specific genetic and expression differentiation that is consistent with positive selection.

However, increased population-specific genetic and expression differentiation of genes with CNVs may not only be attributed to positive selection, but alternatively to relaxed selective constraint. To disentangle these mechanisms, we examined levels of background selection in genes with and without CNVs. Background selection reduces genetic diversity at linked deleterious sites (Charlesworth et al. 1993), and is therefore weaker in regions with reduced selective constraint. As a result, if genes with CNVs primarily evolve under relaxed selective constraint, then we expect a reduction in their levels of background selection relative to those of genes without CNVs. To determine whether this is the case, we compared distributions of median B values (McVicker et al. 2009) in genes with and without CNVs. We found no significant difference between groups (supplementary fig. 2*A*, Supplementary Material online, P>0.05, two-sample permutation test; see Materials and Methods for details), suggesting that overall levels of selective constraint do not differ between genes with and without CNVs. Further, because $F_{\mathrm{ST}}$ is correlated with background selection (Charlesworth et al. 1997), we performed a follow-up analysis in which we explicitly accounted for background selection when comparing the genetic PBS_4_ of genes with and without CNVs. Specifically, we corrected $F_{\mathrm{ST}}$ for background selection using estimated B values (see supplementary Methods, Supplementary Material online, for derivation) and recalculated the background selection-corrected $F_{\mathrm{ST}}$ and genetic PBS_4_ of each gene. Even after this correction, genetic PBS_4_ is elevated in genes with CNVs (supplementary fig. 2*B*, Supplementary Material online, P<0.001, two-sample permutation test; see Materials and Methods for details). Whereas B values are not perfect measures of selective constraint, particularly for short evolutionary timescales, these findings better support the hypothesis that increased population-specific differentiation in genes with CNVs is due to positive selection than to relaxed selective constraint.

### Relationship of Population-Specific Genetic and Expression Differentiation to Gene Function in Europeans

A natural question that emerges from our study is whether there are functional drivers of population-specific genetic and expression differentiation. In answering this question, it was important to exclude YRI, as it is an outgroup to the three European populations and therefore contains greater overall population-specific genetic and expression differentiation that cannot be polarized. Hence, we only considered TSI, GBR, and FIN populations. To globally assess functional modules contributing to population-specific genetic and expression differentiation in these populations, we utilized annotation data from the GO Consortium (Ashburner et al. 2000; GO Consortium 2018). In particular, GO terms classify genes by their molecular functions, cellular components, and biological processes (Ashburner et al. 2000; GO Consortium 2018). Though GO terms refer to intracellular gene functions that cannot be directly related to phenotypes that natural selection acts on, they can aid in elucidating the classes of gene functions that may be associated with population-specific genetic and expression differentiation. To examine these associations, we ranked genes by their genetic and expression PBS_4_ values in each European population, performed GO enrichment analysis on ranked lists, and extracted significantly overrepresented GO terms (supplementary tables S6–S14, Supplementary Material online; see Materials and Methods for details).

After correcting for multiple testing, there are no significantly enriched GO terms for genetic PBS_4_ in any of the populations (supplementary tables S6–S8, Supplementary Material online). However, there are many significantly enriched GO terms for expression PBS_4_ in all three populations (supplementary tables S9–S14, Supplementary Material online). Enriched GO terms for expression PBS_4_ calculated from $P_{\mathrm{ST}}$ with $h^{2}=0.5$ and $h^{2}=1$ are similar, consistent with our previous comparisons (see figs. 1 and 3). Moreover, several enriched GO terms are shared among the three related populations, and numerous related terms are enriched in individual populations. Though most GO terms are quite general and have limited interpretability, it appears that population-specific expression differentiation in Europeans often affects genes involved in signal transduction and immunity. This is not surprising, as such processes are frequent targets of natural selection (Barreiro and Quintana-Murci 2010; Fumagalli et al. 2011; Enard et al. 2016).

To glean further insight into the individual genes potentially driving population-specific genetic and expression differentiation in Europeans, we performed literature searches on genes with the largest genetic and expression PBS_4_ values in each population (tables 1 and 2). In both TSI and GBR, the gene with the largest genetic PBS_4_ value is *MCM6*, or Minichromosome Maintenance Complex Component 6. *MCM6* is part of a protein complex essential for the initiation of eukaryotic genome replication (Labib et al. 2000). Two of its introns contain enhancers for its upstream gene *LCT*, or Lactase, one of which has a mutation prevalent in European populations that is thought to confer lactose tolerance in adulthood (Enattah et al. 2002; Troelsen et al. 2003). Interestingly, *LCT* also has the second-largest genetic PBS_4_ in GBR, and several genetic studies have identified both *MCM6* and *LCT* as targets of recent positive selection in Europeans (Bersaglieri et al. 2004; Voight et al. 2006; Ranciaro et al. 2014; Cheng et al. 2017). In FIN, the gene with the largest genetic PBS_4_ value is *HLA-DPA1*, or Major Histocompatibility Complex, Class II, DP Alpha 1. As a member of the *HLA* gene family, *HLA-DPA1* plays an important role in antigen presentation (Bottazzo et al. 1983) and is believed to be evolving under balancing selection in humans (Hughes and Nei 1988, 1989; Takahata and Nei 1990; Hughes and Yeager 1998; Yasukochi and Satta 2013).

**Table 1:** Genes with Top Five Genetic PBS_4_ Values in TSI, GBR, and FIN

	TSI	GBR	FIN
1	*MCM6*	*MCM6*	*HLA-DPA1*
2	*DCUN1D4*	*LCT*	*RNF114*
3	*DARS*	*CCNT2*	*TRIM47*
4	*CCNT2*	*R3HDM1*	*HSPA2*
5	*PRDM4*	*ZNF615*	*FAHD2B*

**Table 2:** Genes with Top Five Expression PBS_4_ Values ($P_{\mathrm{ST}}$ with $h^{2}=0.5$ and $h^{2}=1$) in TSI, GBR, and FIN

	TSI		GBR		FIN	
	$h^{2}=0.5$	$h^{2}=1$	$h^{2}=0.5$	$h^{2}=1$	$h^{2}=0.5$	$h^{2}=1$
1	*PRKCB*	*PRKCB*	*PRRX1*	*PRRX1*	*VDR*	*FZD1*
2	*TBC1D1*	*TBC1D1*	*CD28*	*CD28*	*FZD1*	*VDR*
3	*BMPR1A*	*KLF3*	*MOB1B*	*INSR*	*TMEM144*	*PLAC8*
4	*KLF3*	*MGAT5*	*BTBD3*	*BTBD3*	*ACTN1*	*FAM134B*
5	*MGAT5*	*FAM65B*	*GLDC*	*TBXT*	*PLAC8*	*SYNJ2*

In TSI, the gene with the largest expression PBS_4_ value (calculated from $P_{\mathrm{ST}}$ with $h^{2}=0.5$ and $h^{2}=1$) is *PRKCB*, or Protein Kinase C Beta. *PRKCB* is involved in numerous signaling pathways, including apoptosis (Reyland 2009) and B cell activation during immune response (Lutzny et al. 2013). As a result, mutations in *PRKCB* are associated with many cancers (Lutzny et al. 2013; Wallace et al. 2014; Antal et al. 2015) and autoimmune diseases (Han, Zheng, et al. 2009; Sheng et al. 2011; Kawashima et al. 2017). The association with autoimmune diseases is particularly intriguing, as such genes are often targets of recent positive selection (Barreiro and Quintana-Murci 2010; Ramos et al. 2014). It is hypothesized that mutations that cause autoimmune response today may have conferred pathogen resistance in the past (Barreiro and Quintana-Murci 2010). In GBR, the gene with the largest expression PBS_4_ value (calculated from $P_{\mathrm{ST}}$ with $h^{2}=0.5$ and$\;h^{2}=1$) is *PRRX1*, or Paired Related Homeobox 1. *PRRX1* is a DNA-associated protein that is involved in the establishment of diverse mesodermal muscle types during development (Martin et al. 1995). It has also been connected with numerous cancers (Takahashi et al. 2013; Guo et al. 2015; Hirata et al. 2015; Jurecekova et al. 2016; Takano et al. 2016; Zhu et al. 2017) and is thought to mediate metastasis (Ocaña et al. 2012; Takahashi et al. 2013; Guo et al. 2015; Zhu et al. 2017). In FIN, the genes with the two largest expression PBS_4_ values are *VDR* followed by *FZD1* when $P_{\mathrm{ST}}$ was calculated with $h^{2}=0.5$, and *FZD1* followed by *VDR* when $P_{\mathrm{ST}}$ was calculated with $h^{2}=1$. *VDR*, or Vitamin D Receptor, interacts with vitamin D in the small intestine to facilitate calcium transportation into circulation (Holick 2006). Skin exposure to solar ultraviolet radiation produces about 90% of the vitamin D that an individual requires (Holick 2006), and living at high latitudes has been associated with vitamin D deficiency due to decreased ultraviolet radiation (Kimlin 2008; Chaplin and Jablonski 2009). Therefore, it is possible that expression differentiation of *VDR* may contribute to high latitude adaptation in FIN. *FZD1*, or Frizzled Class Receptor 1, is a receptor for Wnt signaling proteins (Kennerdell and Carthew 1998). It has been associated with several cancers (Kirikoshi et al. 2001; Benhaj et al. 2006; Zhang et al. 2015) and specifically with chemoresistance (Flahaut et al. 2009), thus making it a promising therapeutic target.

## Materials and Methods

### Gene Expression Analyses

We obtained RNA-seq data from lymphoblastoid cell lines in TSI, GBR, FIN, and YRI populations from the GEUVADIS project (Lappalainen et al. 2013). These data comprise 93 individuals in TSI, 94 individuals in GBR, 95 individuals in FIN, and 89 individuals in YRI, all of whom are from the 1000 Genomes Project (1000 Genomes Project Consortium 2015). We excluded data from the population of Utah Residents with Northern and Western European Ancestry (CEU) because they were collected from an older cell line and have been shown to display expression patterns that are inconsistent with their relationships to other populations (Yuan et al. 2015). We quantified the abundance of transcripts using featureCounts (Liao et al. 2014) with default parameters and the GRCh37 human genome (Zerbino et al. 2018) as our reference. To normalize count data, we used the “median ratio” method (Anders and Huber 2010) by implementing the estimateSizeFactors function in DESeq2 (Love et al. 2014). Next, we calculated the Fragments Per Kilobase of transcript per Million mapped reads (FPKM) of each gene using DESeq2 (Love et al. 2014). We removed genes that contained fewer than ten reads in each sample (lowly expressed), were located on sex chromosomes, or were not protein coding. For the remaining 13,075 genes, we log-transformed their FPKM values by log(FPKM + 1). We computed the $P_{\mathrm{ST}}$ for each gene as $P_{\mathrm{ST}}=\frac{\sigma_{\mathrm{between}}^{2}}{\sigma_{\mathrm{between}}^{2}+2{h^{2}\;\sigma }_{\mathrm{within}}^{2}}$ (Leinonen et al. 2006), where $\sigma_{\mathrm{between}}^{2}$ is expression variance between populations, $\sigma_{\mathrm{within}}^{2}$ is expression variance within populations, and $h^{2}$ is heritability. For our analysis, we used $h^{2}=0.5$ and $h^{2}=1$ as was done previously (Leinonen et al. 2006), though we note that the patterns in figure 1 do not change as a function of $h^{2}$. When $h^{2}=1$, $P_{\mathrm{ST}}$ reduces to $Q_{\mathrm{ST}}$ (Spitze 1993), another common metric for differentiation of quantitative traits between populations.

### Population-Genetic Analyses

We downloaded the 1000 Genomes Project phase 3 data set (1000 Genomes Project Consortium 2015) for TSI, GBR, FIN, and YRI populations from ftp://ftp.1000genomes.ebi.ac.uk/vol1/ftp/, last accessed February 12, 2020. To be conservative in our analyses, we only included the 371 individuals also present in the GEUVADIS Project (Lappalainen et al. 2013). After filtering out insertions, deletions, and monomorphic sites, we were left with 30,734,317 biallelic SNPs. Though we used SNPs of all allele frequencies, limiting our analysis to those with minor allele frequencies >0.01 did not alter our findings. We calculated Hudson’s $F_{\mathrm{ST}}$ for each SNP as $F_{\mathrm{ST}}^{\mathrm{Hudson}}=\frac{{\left(p_{1}-p_{2}\right)}^{2}-\;\frac{p_{1}(1-p_{1})}{n_{1}-1}\;-\;\frac{p_{2}(1-p_{2})}{n_{2}-1}}{p_{1}(1-p_{2})+p_{2}(1-p_{1})}\;$(Reynolds et al. 1983; Weir and Cockerham 1984; Bhatia et al. 2013). Then, we combined SNPs within the entire annotated region of each gene and computed the “ratio of averages” for Hudson’s $F_{\mathrm{ST}}$ (Reynolds et al. 1983; Weir and Cockerham 1984; Bhatia et al. 2013). Because negative $F_{\mathrm{ST}}$ values are not defined (Wright 1951) and have no biological interpretation (Akey et al. 2002), we followed the standard of setting all negative $F_{\mathrm{ST}}=0$ (e.g., Nei 1990; Akey et al. 2002).

### Phylogenetic Analyses

To infer population trees, we first constructed gene trees using the NEIGHBOR program in the PHYLIP package (Felsenstein 2005). We constructed gene trees using either $F_{\mathrm{ST}}$ or $P_{\mathrm{ST}}$ as input distances between populations. Application of the UPGMA algorithm in the NEIGHBOR program yielded totals of 12,977 gene trees for $F_{\mathrm{ST}}$ and 13,075 gene trees for $P_{\mathrm{ST}}$. Next, we used gene trees as input for the CONSENSE program in the PHYLIP package (Felsenstein 1993) and obtained rooted population trees supported by the majority of gene trees based on $F_{\mathrm{ST}}$ and $P_{\mathrm{ST}}$. Specifically, the nodes in gene trees are included if they continue to resolve the population tree and do not contradict with more frequently occurring nodes. The number above each node in figure 1 represents its proportion in all gene trees.

### Calculation of PBS_4_

We first computed the genetic or expression distance between populations as *E*_A,__B_= - log [1 - *Z*_ST_(A , B)], following the approach of Cavalli-Sforza (1969), where *Z*_ST_ represents either $F_{\mathrm{ST}}$ or $P_{\mathrm{ST}}\;$between populations *A* and *B*. We used these as input for calculations of genetic and expression PBS_4_ values. Negative branch lengths were set to 0.

### Gene Ontology Enrichment Analyses

Genes were ranked by their genetic PBS_4_ and expression PBS_4_ values in each population (provided in supplementary tables S3–S5, Supplementary Material online). We performed Gene Ontology (GO) enrichment analysis on each ranked list of genes with the web-based GOrilla tool at http://cbl-gorilla.cs.technion.ac.il/; last accessed February 12, 2020 (Eden et al. 2007, 2009), which searches for enriched GO terms that appear densely at the top of a ranked list of genes (Eden et al. 2007, 2009). For each run, we chose “*Homo sapiens*” as the organism, set the running mode to “Single ranked list of genes,” selected all ontologies (process, function, and component), and set the threshold $P=10^{-3}$.

### Statistical Analyses

All statistical analyses were performed in the R software environment (R Core Team 2013). Two-sample permutation tests were used to assess differences between all pairs of distributions compared in figure 3 and supplementary figures 1 and 2, Supplementary Material online. For each test, we performed 1,000 permutations, using the difference between medians of groups as the test statistic. In particular, we computed the difference between the medians of the two groups for each permutation, and the *P* value of the permutation test as the proportion of times the absolute value of this difference was greater than or equal to the absolute value of the observed difference in the data. Student’s *t*-tests were used to assess the statistical significance of correlation coefficients shown in supplementary tables 1 and 2, Supplementary Material online.

## Discussion

Identifying drivers of human phenotypic differentiation is crucial to understanding adaptive events that occurred in the past, as well as to developing population- and individual-targeted treatments for diseases in the future (Jorde et al. 2001; Sabeti et al. 2002; Akey et al. 2004). Though previous research (Sabeti et al. 2002; Akey et al. 2004; Voight et al. 2006) has made use of abundant whole-genome and polymorphism data for many human populations (International HapMap 3 Consortium 2010; 1000 Genomes Project Consortium 2015) to answer this question, simultaneously studying genetic and expression differentiation may provide unique insights into direct phenotypic targets of natural selection. In particular, it is thought that phenotypic evolution more often occurs through changes in gene regulation and expression, rather than their protein-coding sequences (King and Wilson 1975; Wang et al. 1996; Wray et al. 2003; Carroll 2005, 2008; Raj et al. 2010). For this reason, gene expression differentiation might better reflect phenotypic differentiation. Therefore, a major advantage of the present study is that we utilized both genetic and expression data to address questions about population-specific differentiation in humans. Further, results from our combined analysis suggest that population-specific genetic and expression differentiation in humans may be attributed to several important biological processes, most notably signal transduction and immunity, and also pinpoint many candidate genes for future studies of human phenotypic variation in adaptation and disease.

Yet, there are three key limitations of the data analyzed here that must be considered when interpreting our findings in the context of human evolution. The first is that there is only a single estimate of the expression level of a gene in each population, which is particularly problematic given the complex and dynamic nature of gene expression data. In contrast, there are multiple SNPs per gene in each population, and genetic data are static. Therefore, we expect our estimates derived from expression data to have lower accuracy and higher variance than those from genetic data. Indeed, we found that gene trees constructed with $F_{\mathrm{ST}}$ match the topology of the inferred population tree more often than those constructed with $P_{\mathrm{ST}}$ and, further, that mismatches between topologies of gene trees constructed with $F_{\mathrm{ST}}$ and the inferred population tree are associated with fewer SNPs. Hence, it is also not surprising that genetic and expression PBS_4_ do not have common outlier genes (supplementary tables S3–S5, Supplementary Material online), and gene-level values of expression (and in some cases genetic) PBS_4_ should thus be interpreted with caution. In spite of this issue, a handful of genes with the largest expression PBS_4_ are well-known candidates of adaptation, such as *VDR* (Kimlin 2008; Chaplin and Jablonski 2009). Moreover, at a genome-wide level, the discordance between findings derived from genetic and expression data illustrates the importance of integrating both types of data into population-genetic studies. Nevertheless, future availability of larger sample sizes for gene expression data in multiple human populations will be invaluable for accurately pinpointing genic targets of population-specific expression differentiation in humans.

The second caveat is that TSI, GBR, and FIN are closely related European populations. As a result, genetic distances among them are small, which can lead to noise in gene-level analyses. Moreover, due to shared ancestry and gene flow among these closely related populations, their genetic and expression differentiation are likely to be correlated. This limitation is clearly demonstrated by *MCM6* having the largest genetic PBS_4_ value in both TSI and GBR, which are the most closely related of the three European populations studied. Thus, though genome-wide patterns of genetic and expression differentiation are consistent with population relationships, caution needs to be taken when making inferences based on the genetic and expression PBS_4_ values of individual genes. Despite this limitation, several genes with the largest genetic PBS_4 _values, such as *MCM6* and *HLA-DPA1*, are well-established targets of natural selection (Hughes and Nei 1988, 1989; Takahata and Nei 1990; Hughes and Yeager 1998; Bersaglieri et al. 2004; Voight et al. 2006; Yasukochi and Satta 2013; Ranciaro et al. 2014; Cheng et al. 2017), and novel candidates therefore may represent promising avenues for future research. Nevertheless, phenotypic differences among distantly related populations are better described than those among closely related populations, making it inherently more difficult to interpret our findings in the context of human phenotypes. Therefore, future availability of RNA-seq data from additional populations, particularly those that are more distantly related, will be critical to studying population-specific variation and its role in both human evolution and disease.

The third limitation is that the RNA-seq data used in this study were obtained from lymphoblastoid cell lines. In particular, the enrichment of immune-related functions in genes with high levels of population-specific expression differentiation may be attributed to usage of this cell line, rather than reflecting widespread evolutionary patterns of immunity genes across tissues. Yet, it is important to note that associations between increased population-specific expression differentiation and immunity are consistent with previous findings. Specifically, immunity genes are among the fastest evolving genes in the human genome, likely due to adaptations to rapidly changing environments and introductions of novel pathogens (Barreiro and Quintana-Murci 2010; Fumagalli et al. 2011; Enard et al. 2016). Therefore, though observed patterns of population-specific expression differentiation may not be representative of those in other cell types, genes with high population-specific expression differentiation should be further studied to examine their potential roles in human evolutionary history and disease. Regardless, future availability of RNA-seq data for multiple cell or tissue types in several populations will be invaluable for capturing complex patterns of population-specific expression differentiation and pinpointing genic targets of phenotypic variation among human populations.

In spite of the noted issues with the data analyzed here, a major advantage of our study is the design of PBS_4_, a novel summary statistic that can be used to estimate population-specific differentiation of a quantitative trait in four populations. PBS_4_ requires minimal assumptions about the data and can be used to rapidly estimate population-specific differentiation on a genome-wide scale. Further, because PBS_4_ utilizes data from four populations, branch lengths are more likely to represent true population-specific differentiation than differentiation that occurred ancestral to two populations, as is possible in a three-population scenario (Assis 2019). Therefore, though the data set used in our study is not ideal in many respects, PBS_4_ can easily be applied to existing or future data sets to estimate population-specific differentiation of a wide array of genetic, expression, and other measurable traits in humans and other species. In particular, we envision that application of PBS_4_ to future human RNA-seq data from multiple cell lines or tissues and in many populations of varying divergence levels will shed light on complex questions about human evolutionary history and disease processes.

## Supplementary Material

Supplementary data are available at *Genome Biology and Evolution* online.

## Supplementary Material

evaa021_Supplementary_Data

## Notes

**Data deposition:** This project has been deposited at (https://github.com/xueyuanj/human_PBS; last accessed February 12, 2020). In addition, the GitHub page contains all scripts used in the described analyses, as well as a README file explaining their usage.
