# Supplementary material for: Population-Specific Genetic and Expression Differentiation in Europeans
Source: Genome Biol Evol. 2020 Feb 6;12(4):358–69. doi: 10.1093/gbe/evaa021 (PMC7197493; doi:10.1093/gbe/evaa021)
Supplement: evaa021_Supplementary_Data [file evaa021_supplementary_data.zip › Jiang_Assis_2019_GBE.SupplementaryMethodsFigures_R1.docx]

**Supplementary Methods**

***Correcting*** $\boldsymbol{F}_{\mathbf{ST}}$ ***for background selection using estimated*** $\boldsymbol{B}$ ***values***

A $B$ value measures the drop in local effective population size due to background selection, and can be written as $B=N_{\mathrm{BGS}}/N$, where $N$ is the neutral diploid effective population size and $N_{\mathrm{BGS}}$ is the effective population size under background selection. It follows that the effective population size under background selection is $N_{\mathrm{BGS}}=NB$, or the neutral effective size reduced proportional to $B$. Consider a simple population split model, in which a pair of populations split $\tau_{\mathrm{gen}}$ generations ago, with constant diploid sizes of $NB$ in the ancestral and the descendant populations.

Slatkin (1991) demonstrated that $F_{\mathrm{ST}}$ can be written in terms of coalescence times as

$$F_{\mathrm{ST}}=\frac{\bar{t}-\bar{t}_{w}}{\bar{t}},$$

where $\bar{t}_{w}$ is the expected coalescence time within populations, and where $\bar{t}$ is the expected coalescence time for any pair of lineages, regardless of which population they were sampled in. Thus, $\bar{t}=\frac{1}{2}\bar{t}_{b}+\frac{1}{2}\bar{t}_{w}$, where $\bar{t}_{b}$ is the expected coalescence time between populations. Plugging in gives

$$F_{ST}=\frac{\bar{t}_{b}-\bar{t}_{w}}{\bar{t}_{b}+\bar{t}_{w}}.$$

In our demographic model of constant size of $NB$ diploids and a population split time of $\tau_{\mathrm{gen}}$ generations, we have $\bar{t}_{w}^{\mathrm{gen}}=2NB$ generations and $\bar{t}_{b}^{\mathrm{gen}}=\tau_{\mathrm{gen}}+2NB$ generations. Scaling time in coalescent units of $2N$ generations, we have $\bar{t}_{w}=B$ coalescent units and $\bar{t}_{b}=\tau+B$ coalescent units. Therefore,

$$F_{\mathrm{ST}}(B)=\frac{\tau}{\tau+2B}$$

can be written as a function of the divergence time in coalescent units between the populations ($\tau$) and the $B$ value ($B$).

Under neutrality, we have $B=1$, and hence

$$F_{\mathrm{ST}}=F_{\mathrm{ST}}\left( 1 \right)=\frac{\tau}{\tau+2}.$$

Note that we write $F_{\mathrm{ST}}$ under neutrality by solving for $F_{\mathrm{ST}}$ in the expression

$$\frac{F_{\mathrm{ST}}}{F_{\mathrm{ST}}(B)}=\frac{\tau}{\tau+2}\times\left( \frac{\tau}{\tau+2B} \right)^{-1}=\frac{\tau+2B}{\tau+2}.$$

We can therefore write $F_{\mathrm{ST}}$ under neutrality as

$$F_{\mathrm{ST}}=\frac{\tau+2B}{\tau+2}F_{\mathrm{ST}}\left( B \right).$$

Now, suppose that at each gene we have an estimated $F_{\mathrm{ST}}$ value and its estimated $B$ value (the median $B$ value of the gene). That is, for each gene we have a pair of estimates $\hat{F}_{\mathrm{ST}}(B)$ and $\hat{B}$. We can then *correct* for background selection by plugging the estimated values into the above equation and estimating

$$\hat{F}_{\mathrm{ST}}=\frac{\tau+2\hat{B}}{\tau+2}\hat{F}_{\mathrm{ST}}\left( B \right).$$

However, the issue is that we do not know the divergence time $\tau$. Therefore, we need a mechanism for estimating the divergence time, which should be constant across all genes, as it is the divergence time of the population pair. We can rearrange $F_{\mathrm{ST}}\left( B \right)=\tau/(\tau+2B)$ as

$$B=\left( \frac{1-F_{\mathrm{ST}}(B)}{2F_{\mathrm{ST}}(B)} \right)\tau,$$

and plugging in the estimated $B$ value and estimated $F_{ST}$ for a gene gives

$$\hat{B}=\left( \frac{1-\hat{F}_{\mathrm{ST}}(B)}{2\hat{F}_{\mathrm{ST}}(B)} \right)\tau=\hat{X}\tau,$$

where

$$\hat{X}=\frac{1-\hat{F}_{\mathrm{ST}}(B)}{2\hat{F}_{\mathrm{ST}}(B)}.$$

This is just a linear model, with dependent variable $\hat{B}$, independent variable $\hat{X}$, and slope (or regression coefficient) $\tau$.

Suppose that we have estimates on $K$ genes, with $\hat{B}_{k}$ and $\hat{X}_{k}$ the estimates of $\hat{B}$ and $\hat{X}$ for gene $k$, $k=1,2,\ldots,K$, respectively. Therefore, we estimate $\hat{\tau}$ from entire set of $K$ genes by performing linear regression through the origin on a set of $K$ observations where observation $k$, $k=1,2,\ldots,K$, is $(\hat{X}_{k},\hat{B}_{k})$. Given this estimated divergence time $\hat{\tau}$, we correct for background selection by estimating neutral $F_{\mathrm{ST}}$ as

$$\hat{F}_{\mathrm{ST}}=\frac{\hat{\tau}+2\hat{B}}{\hat{\tau}+2}\hat{F}_{\mathrm{ST}}\left( B \right).$$

Slatkin M. Inbreeding coefficients and coalescence times. 1991. *Genet Res* 58:167-175.


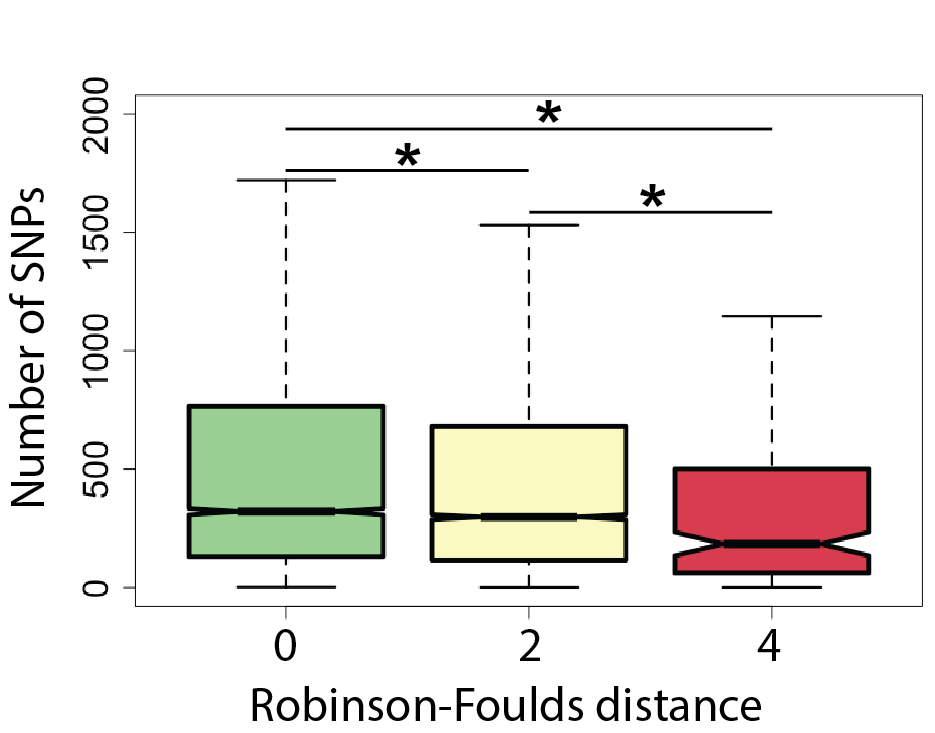


**Supplementary Figure 1. Relationship between number of SNPs used to estimate** $\boldsymbol{F}_{\mathbf{ST}}$ **and differences between constructed gene trees and the population tree.** Distributions of numbers of SNPs used to estimate $F_{\mathrm{ST}}$are shown for Robinson-Founds distances of $RF=0$ (green)$, RF=2$ (yellow), and $RF=4$(red) between gene trees constructed from $F_{\mathrm{ST}}$ and the population tree. *$P<0.01$ (see Materials and Methods for details).

**
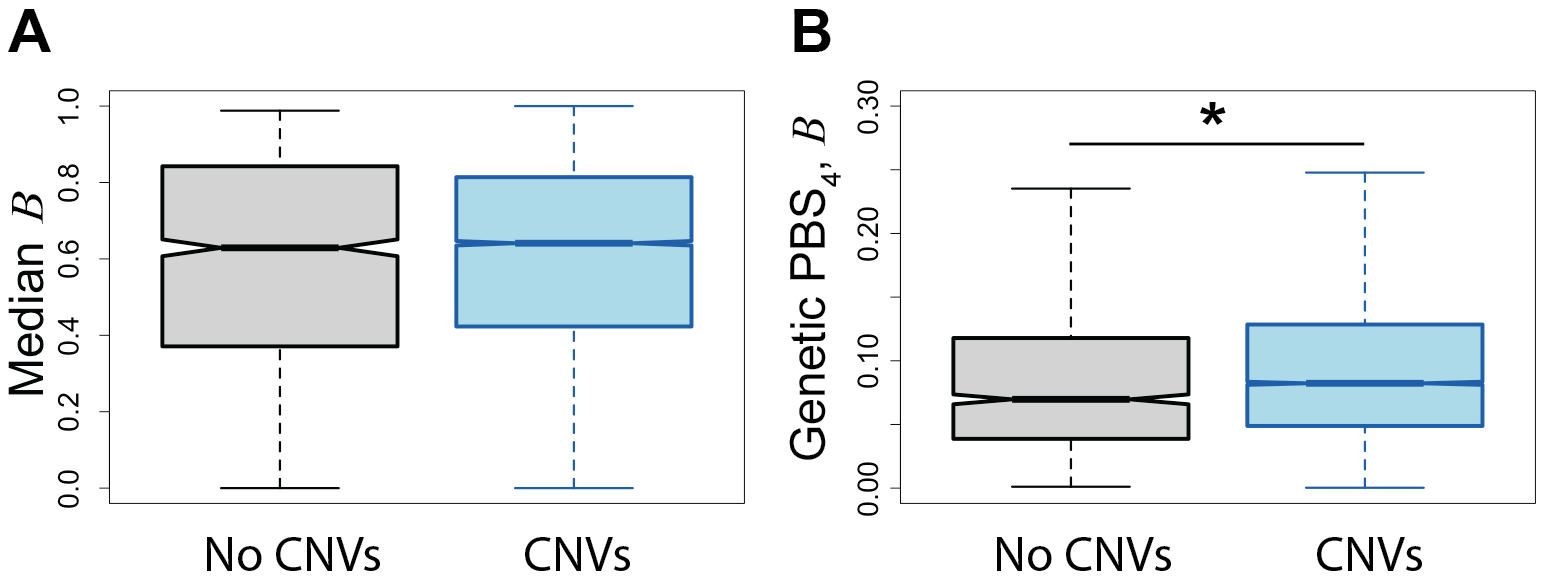
**

**Supplementary Figure 2. Effects of background selection on genetic PBS_4_ of genes with CNVs.** Distributions of (*A*) median $B$ values and (*B*) genetic PBS_4_ after correcting $F_{\mathrm{ST}}$ for $B$ of genes without (gray) and with (blue) CNVs. *$P<0.001$ (see Materials and Methods for details).
