# Supplementary material for: Population-Specific Genetic and Expression Differentiation in Europeans
Source: Genome Biol Evol. 2020 Feb 6;12(4):358–69. doi: 10.1093/gbe/evaa021 (PMC7197493; doi:10.1093/gbe/evaa021)
Supplement: evaa021_Supplementary_Data [file evaa021_supplementary_data.zip › Jiang_Assis_2019_GBE.Supporting_Information.docx]

**Supplementary Figure Legends**

**Supplementary Figure 1. Relationship between number of SNPs used to estimate** $\boldsymbol{F}_{\mathbf{ST}}$ **and differences between constructed gene trees and the population tree.** Distributions of numbers of SNPs used to estimate $F_{\mathrm{ST}}$are shown for Robinson-Founds distances of $RF=0$ (green)$, RF=2$ (yellow), and $RF=4$(red) between gene trees constructed from $F_{\mathrm{ST}}$ and the population tree. *$P<0.01$ (see Materials and Methods for details).

**Supplementary Figure 2. Effects of background selection on genetic PBS_4_ of genes with CNVs.** Distributions of (*A*) median $B$ values and (*B*) genetic PBS_4_ after correcting $F_{\mathrm{ST}}$ for $B$ of genes without (gray) and with (blue) CNVs. *$P<0.001$ (see Materials and Methods for details).
