# Supplementary figures and images for: Population-Specific Genetic and Expression Differentiation in Europeans
Source: Genome Biol Evol. 2020 Feb 6;12(4):358–69. doi: 10.1093/gbe/evaa021 (PMC7197493; doi:10.1093/gbe/evaa021)

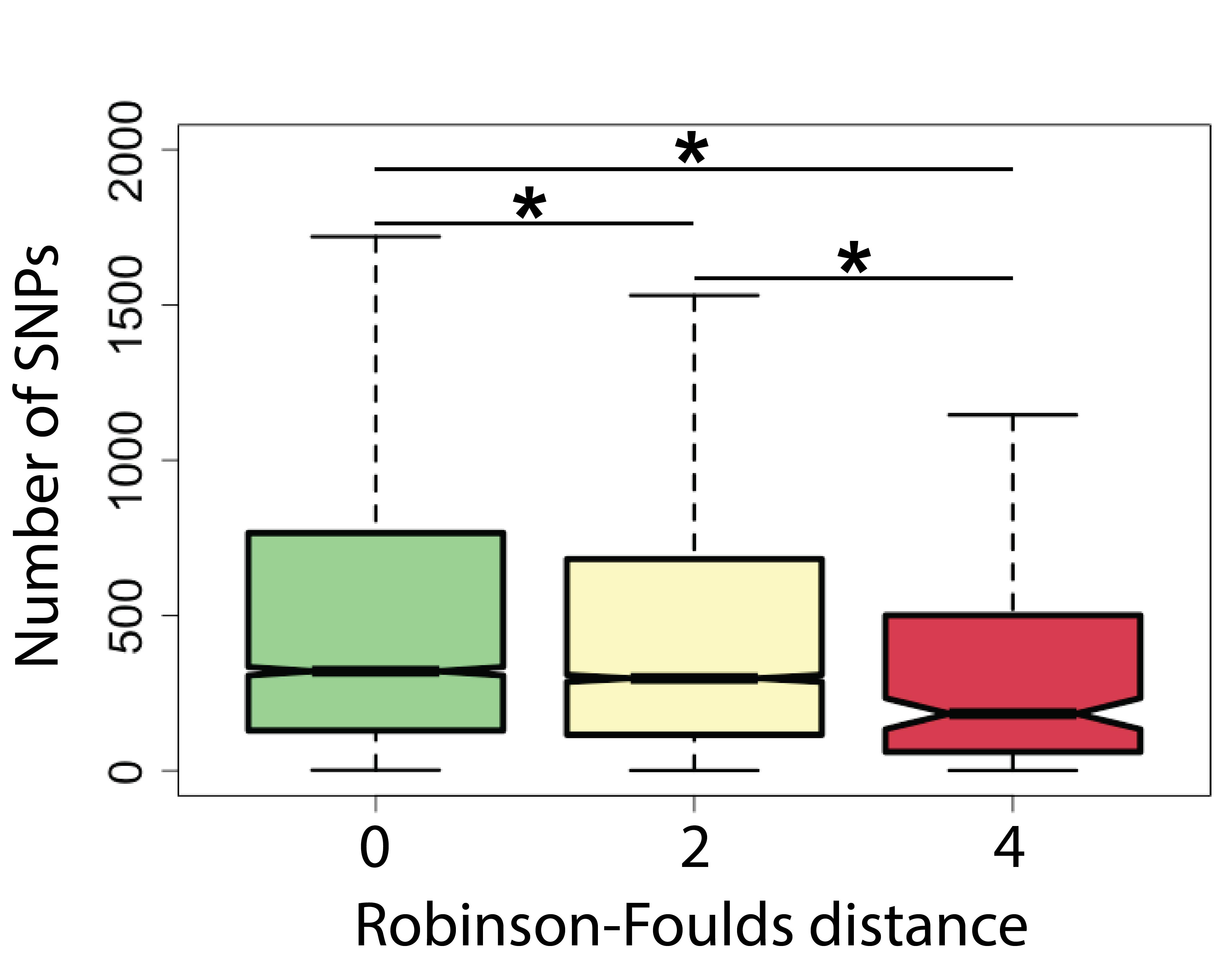

Supplement: evaa021_Supplementary_Data [file evaa021_supplementary_data.zip › Fig.S1.jpg]

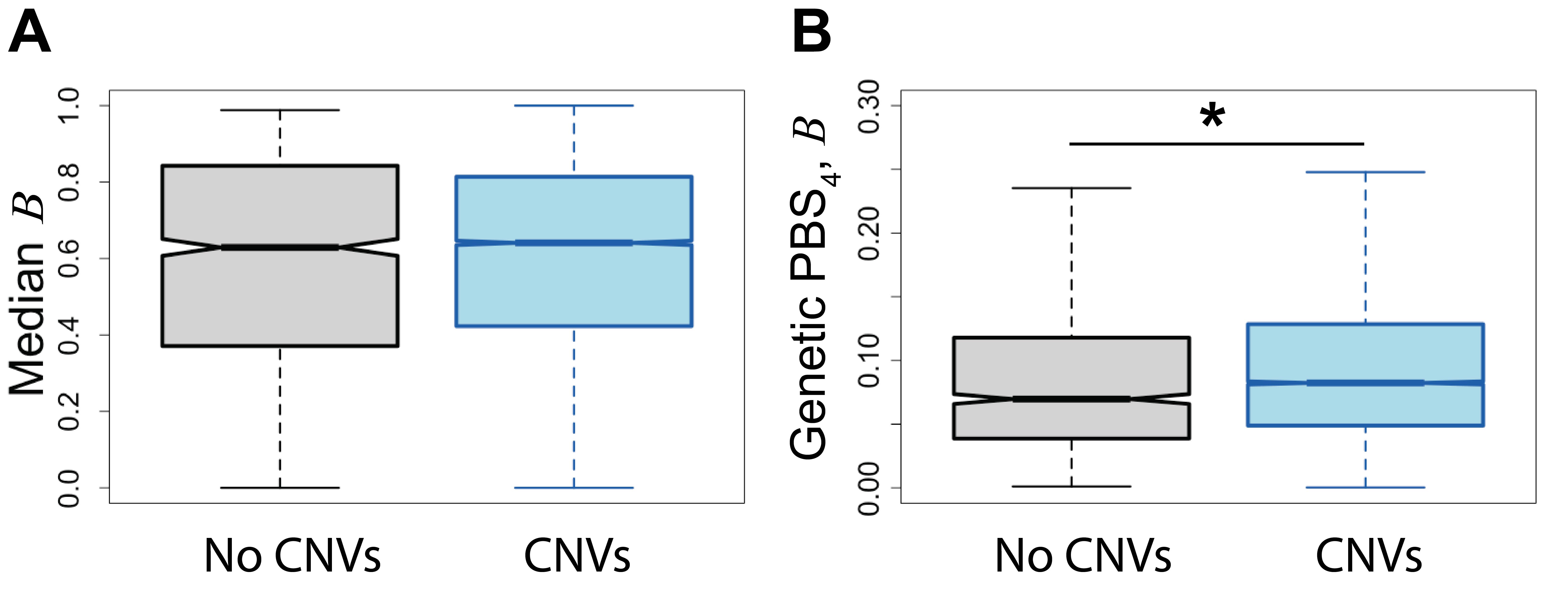

Supplement: evaa021_Supplementary_Data [file evaa021_supplementary_data.zip › Fig.S2.jpg]
